# Measurement of the potential geographic accessibility from call to definitive care for patient with acute stroke

**DOI:** 10.1186/s12942-018-0121-4

**Published:** 2018-01-12

**Authors:** J. Freyssenge, F. Renard, A. M. Schott, L. Derex, N. Nighoghossian, K. Tazarourte, C. El Khoury

**Affiliations:** 1 Univ. Lyon, University Claude Bernard Lyon 1, HESPER EA 7425, 69008 Lyon, France; 2 Emergency Department and RESCUe Network, Lucien Hussel Hospital, Vienne, 38200 France; 3 UMR 5600 Environnement Ville Société CNRS, University Jean Moulin Lyon 3, 18, rue Chevreul, 69007 Lyon, France; 4 Pôle IMER, Hospices Civils de Lyon, 69003 Lyon, France; 5 Department of Stroke Medicine, Hospices Civils de Lyon, 69003 Lyon, France; 6 Department of Neuroradiology, Hospices Civils de Lyon, 69003 Lyon, France; 7 CREATIS, CNRS-UMR5220 INSERM-U1044, Lyon, 69008 France; 8 INSA-Lyon, Lyon, 69008 France; 9 Emergency Department, Hospices Civils de Lyon, 69003 Lyon, France

**Keywords:** Stroke, Geographic accessibility, Road network, Medical transport, Health services

## Abstract

**Background:**

The World Health Organization refers to stroke, the second most frequent cause of death in the world, in terms of pandemic. Present treatments are only effective within precise time windows. Only 10% of thrombolysis patients are eligible. Late assessment of the patient resulting from admission and lack of knowledge of the symptoms is the main explanation of lack of eligibility.

**Methods:**

The aim is the measurement of the time of access to treatment facilities for stroke victims, using ambulances (firemen ambulances or EMS ambulances) and private car. The method proposed analyses the potential geographic accessibility of stroke care infrastructure in different scenarios. The study allows better considering of the issues inherent to an area: difficult weather conditions, traffic congestion and failure to respect the distance limits of emergency transport.

**Results:**

Depending on the scenario, access times vary considerably within the same commune. For example, between the first and the second scenario for cities in the north of Rhône county, there is a 10 min difference to the nearest Primary Stroke Center (PSC). For the first scenario, 90% of the population is 20 min away of the PSC and 96% for the second scenario. Likewise, depending on the modal vector (fire brigade or emergency medical service), overall accessibility from the emergency call to admission to a Comprehensive Stroke Center (CSC) can vary by as much as 15 min.

**Conclusions:**

The setting up of the various scenarios and modal comparison based on the calculation of overall accessibility makes this a new method for calculating potential access to care facilities. It is important to take into account the specific pathological features and the availability of care facilities for modelling. This method is innovative and recommendable for measuring accessibility in the field of health care. This study makes possible to highlight the patients’ extension of care delays. Thus, this can impact the improvement of patient care and rethink the healthcare organization. Stroke is addressed here but it is applicable to other pathologies.

## Background

Stroke is the main cause of non-traumatic disability and the second major cause of death in the world. WHO (World Health Organization) refers to stroke in terms of pandemic. Indeed, stroke caused 6.7 million deaths in 2012 [1]. Today, the pathology affects one person each second in the world. In 2015, a case of stroke occurred every 4 min in France, with 130,000 full hospitalisations [2]. The financial cost of stroke is high, with an annual 8.4 billion euros of sanitary and medical and social expenditure and an average €9642 per year for patients who have suffered stroke causing invalidity (about 14% more than for a long-term patient with Alzheimer’s disease), covered by state medical insurance [3].

Stroke is a pathology bound to the notion of time. The effectiveness of treatment today is limited to precise temporal windows. For eligible patients, thrombolysis must be performed within 4 h 30 min of the appearance of the symptoms and thrombectomy within 6 h. Only 10% of patients are eligible for thrombolysis. This small proportion would seem to be the result of the late presentation of the patient and poor knowledge of the symptoms [4]. On the principle of ‘time is brain’, the aim of this paper is therefore to measure the access times to treatment facilities for stroke patients. This measurement is assessed using innovative methodology that models access time from the existing road network and using various means of transport.

Assessment of geographic accessibility is essential in many kinds of pathology. With the development and perfecting of Geographic Information Systems (GIS) in recent years, attempts have been made in a number of studies to model this accessibility as the use of a GIS to assess access times has been validated [5, 6]. Two studies have been essential for the development of this approach. The first [6] aimed to demonstrate the advantages of a GIS. Nadine Schuurman et al. [7] mention the ‘array of spatial analysis methods’ offered by GIS, providing ‘an efficient and flexible way’ to examine models of geographic access to health services. The study by Apparicio et al. [8] has also been a solid base for the use of GIS in assessing geographic access to a pathology. Indeed, in this case, the question is that of understanding the use of GIS to calculate accessibility that matches the phenomenon and the area observed as best as possible and that limits clumping errors. From 1980, a large number of articles have been published on the geographical accessibility topic, and this number is growing since the 2000s [9]. In our study, the topic of spatial accessibility is developed, defined like the fusion between accessibility dimension (distance or time between patient and service points) and availability dimension (number of service points from which a patient can choose) by Mark Guagliardo [10]. The measure of spatial accessibility can be classified in four categories: provider-to-population ratios, distance to the nearest provider, average distance to a set of providers, gravitational models of provider influence [10]. In this study, the travel impedance to nearest provider is studied. The travel impedance to nearest provider considers the accessibility but not the availability. Some other methods of spatial accessibility measures take into account these two dimensions [6, 10–12]: the two step floating catchment area method ‘2SFCA’ [13, 14], gravity model [15] and the Kernel density model [10].

In the case of travel impedance to nearest provider [11, 12] a first work on modelling geographic access to emergency departments was conducted in 2009 in the United States [16]. The paper was a precursor as accessibility was addressed in terms of travelling time from the road network at different time steps, making possible the analysis of regional availability of emergency care. However, many non-geographic factors such as traffic congestion or difficult weather conditions (snow, fog, heavy rain, etc.) that disturb traffic were not taken into account and this feature seems to be an important limit to the study. One of the most complete studies so far towards accessibility is that of Alford-Teaster et al. [17] on mammography access. The use of care facilities is examined along with geographic accessibility. The study was performed from the angle of various factors such as demographic and economic criteria and land use. However, movements of patients in vehicles other their own are not shown in the study. Two studies are particularly interesting regarding to stroke. The first, by Scott et al. [18], showed the advantages of GIS for calculating time steps of journeys established according to the identification of symptoms for access to thrombolysis and using the positions of hospitals in Canada with a Stroke Unit. This study is nearly 20 years old. GIS techniques have been improved and perfected and the same modelling would be more representative of areas and the road network and hence more pertinent today. Concerning stroke care, the study of Adeoye et al. [19] seems to be the most complete study on accessibility. It describes access of the US population to hospital stroke care facilities on the basis of transport by medicalised ambulance in every case.

A review of the literature on the accessibility of stroke care facilities shows that no study of this kind had been performed in France. It was therefore essential to perform such analysis to learn more about territorial accessibility in the Rhône area. Furthermore, the limits of previous studies were taken into account—factors that are not inherently geographic such as traffic difficulties and the modelling of different types of transport and transporter. This consideration also made it possible to use different accessibility scenarios to adapt the study to territorial issues.

The aim of this study was to develop a measure method of accessibility according to the localization of patients and the different means of care.

## Methods

### Study area and data sources

**Fig. 1:**
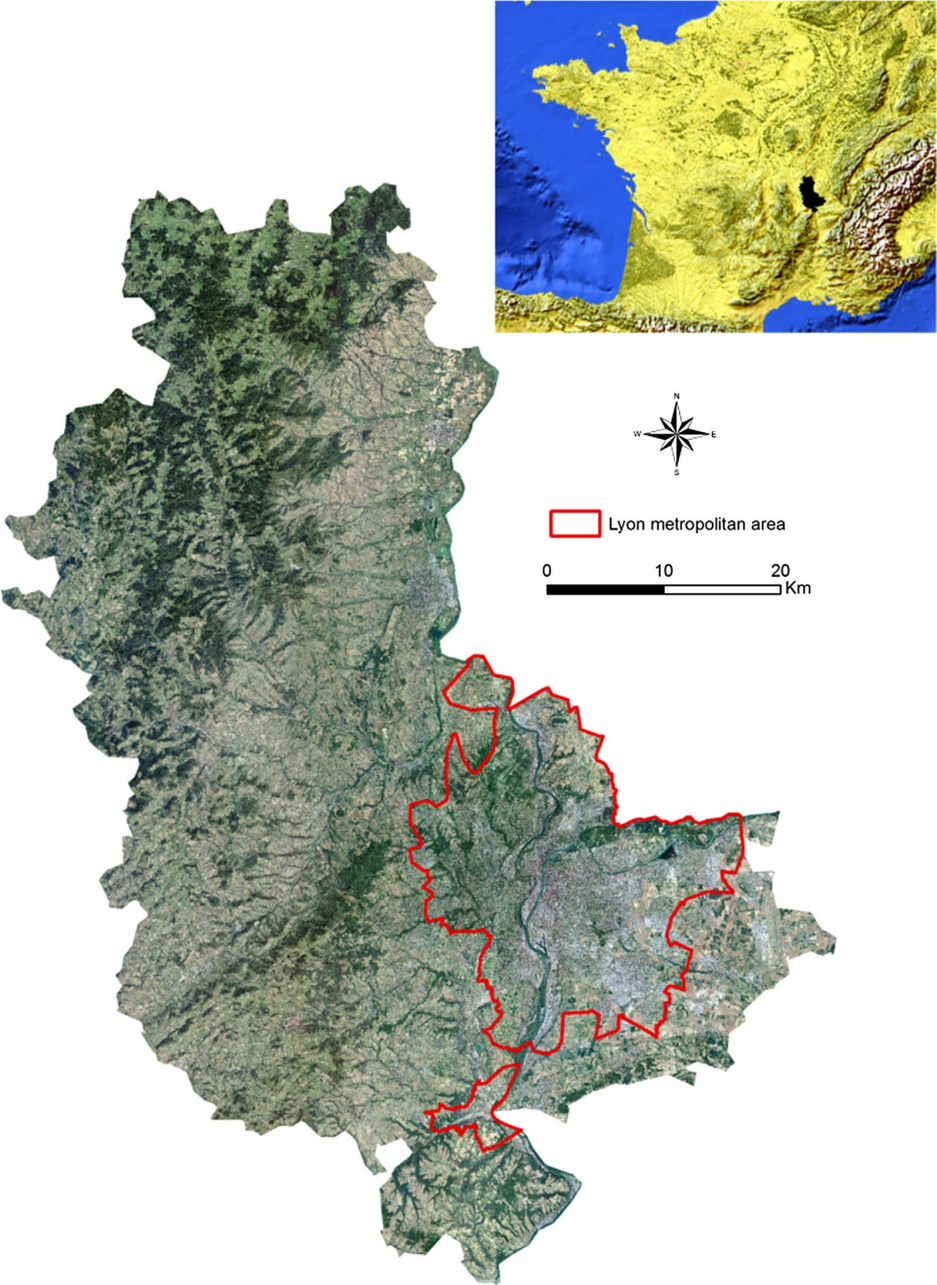
Orthophotograph (resolution 5 m) of the study area and its location in Western Europe (top right)—sources: IGN and Natural Earth

Our study area is the Rhône administrative county in France (Fig. 1). The county covers an area of 3249 km^2^ with a population of 1,798,511 in 2014 (source: *Institut national de la statistique et des études économiques*, Insee), i.e. 543 persons per square kilometre. Persons over 65, known to have a greater risk of stroke [20, 21], form 15.5% of the total population. A tendency for ageing is observed in the county, as is seen in all developed countries [22] as these have completed their demographic transition. Thus the number of over-60 s increased by 12% from 2007 to 2012 whereas the population as a whole increased by only 5%. The Rhône-Alpes region accounted for 9.7% of the French Gross Domestic Product (GDP), making it the second region in France and the seventh in Europe in terms of economic weight [23]. The driving force of the area is the metropolis of Lyon (Lyons), the second largest urban area of France with a population of 1.3 million.

The choice of road network is primordial for modelling transport operations. Description of accessibility using Euclidian distances [24] is a first approach but remains fairly limited. Geographic Information Systems have evolved and became essential to model access according to the road network. In previous French studies [25, 26], measurement of accessibility had used the *Route500* road database of the *Institut national de l’information géographique et forestière* (IGN). This database shows the main 500,000 kilometres of French roads: motorways, national main roads and departmental roads but passes over many residential and urban sections. Therefore, we have chosen to use the IGN *BDCarto* database, with more than a million kilometres of roads—the entire French road network—to get a more complete picture of the territory.

### Pre-hospital emergency system

The ability of a care system to handle acute complications of medical or traumatic pathologies depends on many geographic and spatial criteria, making it a key public health issue. The times needed for access to treatment are a major factor in the care system. Two main types of vital emergency care system coexist in developed countries. The ‘Anglo-Saxon’ system relies on management of prehospital emergency by paramedics in order to transfer the patient to the nearest hospital. In this context, the goal is the fastest transport to medical facility. This system is mostly that of United States, Canada and United Kingdom where most of intensive care is performed at the hospital. The other system relies on emergency physicians involved at the scene of injury. This Emergency Medical Service (EMS) system is effective in France, Greece, Germany, Canada or Austria for example [27]. The ‘French’ system is based on sending to the patient a medical team called a *Unité Mobile Hospitalière*, UMH (a ‘Mobile Hospital Unit’) before entry to a hospital. The mobile unit assesses the seriousness of the case, makes a diagnosis, performs the necessary emergency and therapeutic support and then transfers the patient to a suitable hospital with facilities available—which is not necessarily the nearest one.

In France, the UMHs are part of *Structures Mobile d’Urgence et de Réanimation*, SMUR (Mobile Intensive Care Unit) that are located at public hospitals. Each SMUR is in charge of operational coverage of a defined population area. The segmentation of SMUR sectors depends on administrative boundaries without considering care access times for the population.

The *Service d’Aide Médicale Urgente* or SAMU (Medical Dispatch Call Center) is in charge of the reception and the triage of calls for each SMUR area. It is responsible for all prehospital medical or traumatic emergencies 24 h a day and 7 days a week, using the 15 call. Each centre is equipped with advanced telephone equipment and numerous information systems (geolocalisation, computerised filing, operational listing of resources). The SAMU centres are manned by medical staff, triage emergency doctors, general practitioners and non-medical staff (medical triage assistants).

The work of SAMU units is specified by decree and can be summarised in five broad categories:

- Decide and implement the most suitable response to the call as rapidly as possible,
- Check the availability of the public or private hospitalisation facilities appropriate for the condition of the patient, and ensure that arrangements were made for the arrival of the latter,
- If necessary, organise transport to a public or private hospital, using a public service (the fire brigade) or a private ambulance company,
- Oversee the admission of the patient, involving coordination of on-site care by the SMUR team and the hospital admission service,
- Provide support in the search for a specialised technical facility for non-academic hospitals.

The EMS system in France is not only based on UMH care. In fact, UMHs are the second level of EMS system. The first level is composed of basic life support (BLS) fire department ambulances, based at fire stations [19].

In Rhône county, it is not possible for patient to go directly to the Comprehensive Stroke Center (CSC), with private car. The patients are constantly transported by ambulances (SAMU or firemen ambulance). For stroke care there is a dedicated procedure from the call to EMS to the admission in CSC who not includes transport by private car. In most cases, stroke patients are transported to hospital by firemen because they don’t need medical transport.

### Data analysis

All the cartographic analyses shown in this work were produced using the program ArcMap *10.4.1* [28]. Measurements of accessibility were performed on the territory concerned by the study (the Rhône county) and neighbouring counties. Incorporating the road network in these areas in the model makes it possible to show the border effects associated with neighbouring counties population flows. The logic of movements is therefore evaluated here. Once the study area had been clearly established, it was essential to determine the speed limits allocated to each section according to the *Code de la Route* (Highway Code). As the *BDCarto* database does not include this information, the collaborative database *OpenStreetMap* [29] was used. However, information was lacking for 13% of the segments once the speed limits of the segments had been completed using this database. Therefore, thanks to CORINE Land Cover [30], a European georeferenced vectorial database showing land use, the speed limits were completed where they were lacking in the 13% of sectors. A speed limit of 50 kph (31 mph) was applied in urban zones and 90 kph (56 mph) in rural zones. Noting the speed limit in each section thus means that accessibility can be calculated in terms of time and not just in terms of distance.

#### Modelling according to accessibility scenarios

Various kinds of modelling were conducted with the varying of speed limits to take into account all the territorial issues involved in the Rhône county. The *Network Analyst* extension of *ArcMap 10.4.1* was used for these scenarios [7, 8, 19]. Four scenarios are shown in this study. They depend on traffic conditions and type of transport (Table 1).

**Table 1:** Characteristics of the different scenarios used (French law limits speeds to 130 kph (81 mph) or 110 kph (68 mph) on motorways, 90 kph (56 mph) in rural and periurban areas and 50 kph (31 mph) in built-up areas. However, local special features may be applied according to the context

Scenario	Modelling	Speed	Justification of speed adaptations
Scenario 1	Initial database	Respect of national speed limits	Private car submitted to French Highway Code respect
Scenario 2	Emergency transport	20 kph (12 mph) above the limit throughout the road network	Analysis based on Rhône’s SAMU stroke interventions between 2012 and 2016, and Petzäll et al. [31] study
Scenario 2’	Emergency transport in urban and rural areas	20 kph (12 mph) higher in rural areas and 10 kph (6.2 mph) lower in urban areas	Complementary scenario for sensitivity analysis, based on literature review (Petzäll et al. [31]) and SAMU interventions analysis
Scenario 3	Difficult weather conditions (rain, fog, snow)	20 kph (12 mph) lower than the limit throughout the road network	Respect of the R413-2 article French Highway Code when there is severe weather conditions
Scenario 4	Emergency transport with traffic jams in the Lyons city area	20 kph (12 mph) lower in the Lyons metropolitan network (59 communes), 20 kph (12 mph) higher in the rest of the network	Petzäll et al. [31] study and SAMU interventions analysis for 20 kph higher and SAMU interventions analysis during traffic jam for 20 kph lower

The first scenario is the case of patient transportation by private car, the driver respects the speed limitations. The postulate is the movement of patients by their own means (their own vehicle or with help of a friend or relation) to the closest treatment facility. If the driver of private car decides not to respect the speed limitations, the other scenarios can be applied.

Scenario 2 is for driving in an emergency situation. The following hypothesis is used when modelling tends to show access to stroke care facilities from all points in the network: for private car, a third part transports the patient and takes the decision not to respect the speed limits. Petzäll et al. [31] measured an increase by 21.5 kph (13.3 mph) for emergency transportation by ambulances for cardiovascular disorders, 19.8 kph (12.3 mph) in urban area, 23.2 kph (14.4 mph) in rural area and 21.1 kph (13.1 mph) for extensive need of care, for a study based on the Swedish road network. Furthermore, an analysis based on SAMU interventions has been realized. From 2012 to 2016, the SMUR of Lyon has realized 646 interventions for stroke patients, prehospital medical management of stroke is indicated in case of a coma and concerns about 5% of strokes. From this reported data, the SMUR moves with an average speed of 21.46 kph (13.33 mph) faster than a private care with similar driving conditions. This average speed transport has been calculated from the precise location of SMUR departure center to the centroid of destination city (the precise location of patient care is not reported). According to these statements, the speed limits have been increased by 20 kph (12 mph) for scenario 2.

Scenario 2’ is a complementary scenario which models the accessibility depending on the kind of area. In fact, on the basis of variation times between urban and rural areas [16, 31–33], the speed limitations are always increased by 20 kph (12 mph) in rural areas and decreased by 10 kph (6.2 mph) in urban areas, based on the Petzäll et al. [31] who has measured a speed difference of 30 kph (18.6 mph) between rural and urban areas.

In Scenario 3, with lower speeds due to severe weather conditions, we consider that the driver takes the decision to reduce his speed by 20 kph (12 mph) and thus respect the R413-2 article of French Highway Code recommendations if weather conditions are difficult [34].

The fourth scenario is an adaptation of scenario 2 and analysis of SAMU interventions during traffic jam hours.

#### Cartographic representation

A series of polygons was calculated for the zones covered (isochrones) representing the distance that can be attained from each stroke treatment facility in a particular length of time. The first stage thus consists of listing and geolocalising each stroke treatment infrastructure. Thus, public hospital emergency departments, Primary Stroke Center (PSC) and Comprehensive Stroke Center (CSC) [35] in the Rhône and neighbouring counties were geolocalised using their precise postal addresses (Table 2).

**Table 2:** Distribution of patient admission infrastructure

Infrastructure	County	Staff
Emergency department	Rhône (69)	7
	Ain (01)	3
	Saône-et-Loire (71)	6
	Isère (38)	7
	Loire (42)	6
PSC	Rhône (69)	2
	Ain (01)	1
	Saône-et-Loire (71)	1
	Isère (38)	2
	Loire (42)	2
CSC	Rhône (69)	1
	Ain (01)	0
	Saône-et-Loire (71)	0
	Isère (38)	1
	Loire (42)	1

Preliminary measurement of accessibility to facilities from all points in the network with 10, 20, 30, 45 and 60-min access time was thus calculated. These time steps were chosen after bibliographic analysis [7, 16–19, 36, 37]. Each scenario was applied to this modelling.

**Fig. 2:**
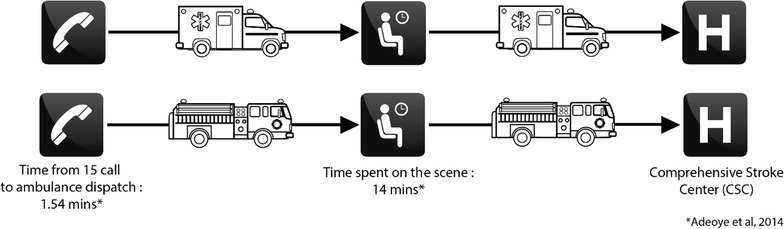
Diagrammatic representation of overall journey time modelled according to the type of transport (SMUR and fire brigade)

Although it is pertinent to characterise the area according to the time required for travel from any point in the network to the treatment facility, it is even more interesting to model overall admission time. Treatment of stroke requires the best possible upstream taking in hand of the patient [4, 38]. This means that it is necessary to know the pattern of the territory according to the type of transport and also the positions of stroke treatment facilities. In our case, the development of thrombectomy and recent studies have shown its advantages for patients [39–41] and modelling overall patient reception was performed using the location of the CSC. With this model it is possible to characterize the territory by care delays from the emergency call to the admission in nearest CSC. It is a global approach of care because all the times of pre-hospital emergency care for stroke patients are taken into account. The second phase of our study was therefore aimed at georeferencing each fire station in the Rhône and neighbouring counties, together with each SMUR team, using their precise addresses. After this georeferencing, supply zones were calculated for these facilities and then for each CSC to finally show total admission time—i.e. the estimated times from SMUR centres or fire stations to all the points in the network and then from any point to the CSC (Fig. 2). In this model, private car is not considered because it is not possible for patient to go directly to the CSC by his own. It was interesting to take intervention and triage times into account to better estimate the time. Thus, after a review of the literature, average time for ambulance dispatch, time spent at the scene and transport to a Comprehensive Stroke Center and intervention at the site of occurrence determined by Adeoye [19] were chosen (Fig. 2). The final times were calculated using the United States EMS (Emergency Medical Service) register for stroke cases alone.

This overall approach was represented on the basis of IRIS area units (*Ilots Regroupés pour l’Information Statistique*, small zones grouped for statistical information), the smallest administrative division of Insee (*Institut National de la Statistique et des Etudes Economiques*) and that respect demographic (populations of 2000) and geographic criteria [42]. This representation gives an accurate view and characterisation of areas according to access time to an CSC. For thrombectomy, discussions are in progress with regard to direct admission to CSC (Mothership) or a first stop at PSC (‘Drip ‘n Ship’) [35]. In this study accessibility has been modelled using the Mothership pattern.

## Results

### Potential accessibility to care facilities in the Rhône county

The various scenarios (Table 1) combined with the diversity of infrastructure density in the area reveal marked differences in accessibility.

**Fig. 3:**
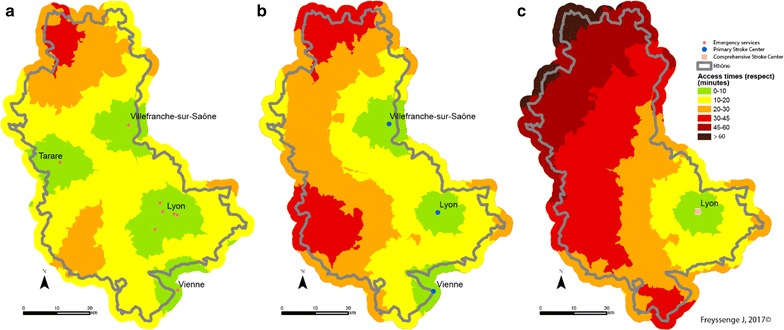
Access to facilities (emergencies: map **a**, PSC **b** and CSC **c**) at all points in the network when speed limits are respected (Scenario 1)

Logically, for each scenario, as there are more emergency services that PSCs, and are much more numerous than CSCs, access times to the latter are shorter. Certain zones in the area are under-privileged, whatever the scenario considered. Indeed, towns in the northern part of the Rhône such as Monsols, Aigueperse and Proprières are at least 30 min from the closest emergency department when speed limits are respected (Fig. 3a). The north of the county is again more than 30 min from an PSC facility. The area concerned is sometimes larger in this case and a zone with travelling times of more than 30 min is seen in a large south-western part: this is the case of the towns Bessenay and Duerne (Fig. 3b). For CSC, more than half of the county is more than 30 min from a facility and the journey from the commune of Aigueperse takes more than 60 min (Fig. 3c).

**Fig. 4:**
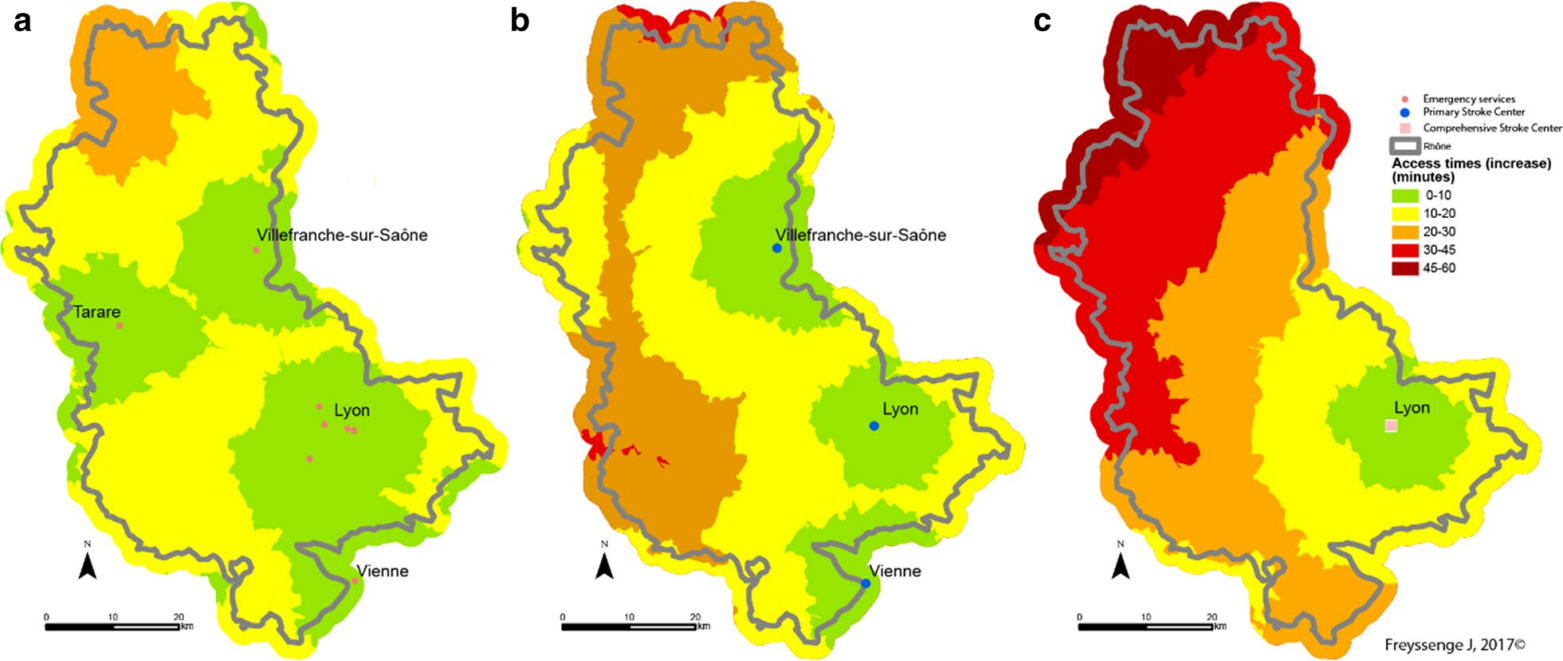
Access to facilities (emergency: map **a**, PSC **b** and CSC **c**) at all points in the network with emergency transport exceeding the speed limits by 20 kph (12 mph) in Scenario 2

**Fig. 5:**
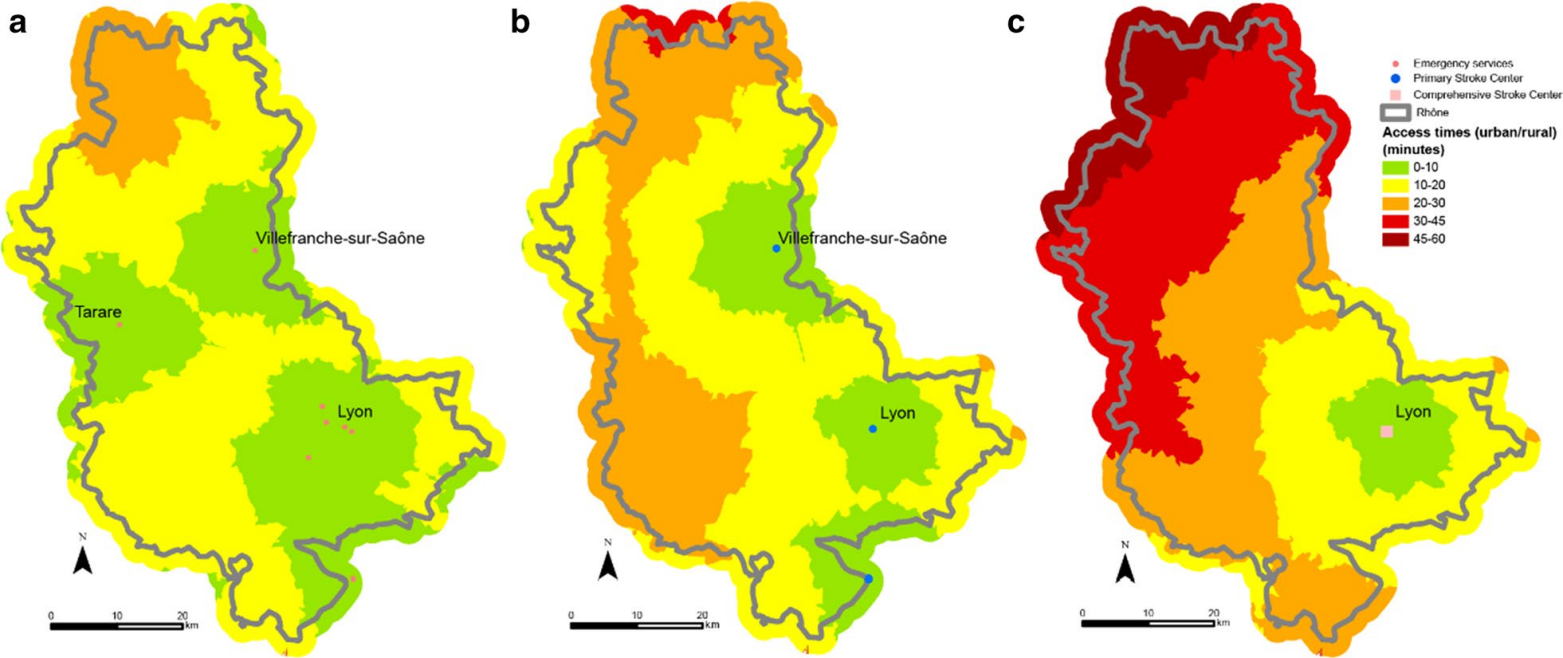
Access to facilities (emergency: map **a** PSC **b** and CSC **c**) at all points in the network with emergency transport exceeding the speed limits by 20 kph (12 mph) in rural areas and 10 kph (6.2 mph) in urban areas below the speed limits in Scenario 2’

When speed limits are not respected with an additional 20 kph for each section (Scenario 2), the entire territory is less than 30 min from the closest emergency department. Similarly, for PSC, with the exception of a very small part of the area (the northern and western extremes), communes in the Rhône county have access in less than 30 min. However, the north-western third of the Rhone area is more than 30 min from the nearest CSC (Fig. 4). There are no major variations with scenario 2’, the service area for PSC and CSC is less extended in urban areas but it is not really significative (Fig. 5).

**Fig. 6:**
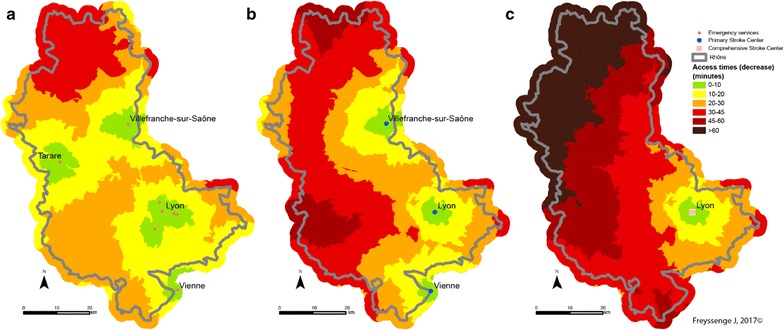
Access to facilities (emergency: map **a** PSC **b** and CSC **c**) at all points in the network with adverse weather conditions (reduction of regulation speed by 20 kph (12 mph) Scenario 3)

The third scenario consists of travelling 20 kph (12 mph) slower than the speed limits because of difficult weather conditions (fog, ice or snow for example). Access time is naturally longer in this case. The northern part of the Rhône—the Beaujolais area in particular—is harder hit once again. This section is more than 30 min from the nearest emergency department. The western and northern parts of the county are thus more than 30 min from a facility and even more than 45 min from a facility when potential access times to the closest PSC units are measured. Few CSC facilities are available in the Rhône and neighbouring counties and potential access time is very varied. Indeed, some areas are distinctly underprivileged. Access to thrombectomy for the populations of the communes concerned is thus affected. Here again, the north-western part of the county has markedly fewer facilities and access times exceed 60 min (Fig. 6).

**Fig. 7:**
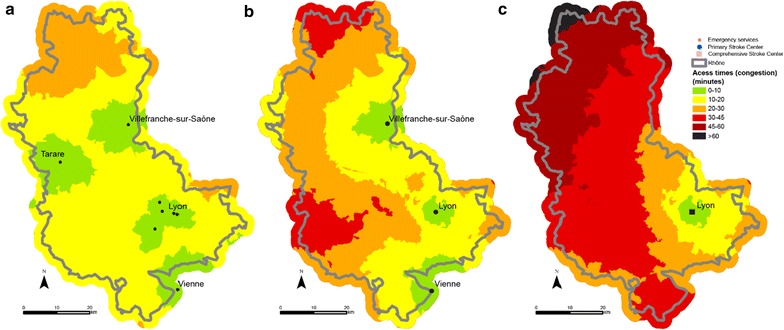
Access to facilities (emergency: map **a** PSC **b** and CSC **c**) at all points in the network during traffic congestion in the Lyons urban area (Scenario 4)

The final scenario showing road congestion does not seem to cause significant changes in access times to the various facilities times except in Greater Lyons. However, as the communes in the metropolis of Lyons are not concerned by major lack of access, traffic jams do not seem to be an obstacle to care (Fig. 7).

**Fig. 8:**
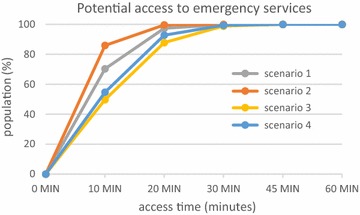
Potential access to emergency services for the population of the Rhône

**Fig. 9:**
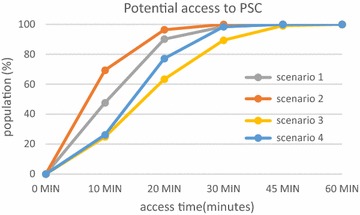
Potential access to PSC for the population of the Rhône

**Fig. 10:**
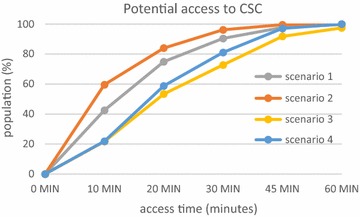
Potential access to interventional neuroradiology (CSC) for the population of the Rhône

The proportion of population with access to facilities in a given time varies considerably. When speed limits are respected (Scenario 1), 98% have 20-min access time to the nearest emergency department (Fig. 8), 90% has 20-min access time to an PSC facility (Fig. 9) and 75% has 20-min access time to an CSC facility (Fig. 10). Thus, virtually a quarter of the population of Rhône county does not have access to CSC in less than 20 min whereas this access time is practically 100% for emergency services. This raises the question of the eligibility of patients for treatment of stroke as we know that patients have little awareness of the symptoms and are often long to call the emergency number (15) or to go to an emergency department. For example, poor accessibility of CSC plays an important role for patients who arrive too late for thrombectomy.

However, access to facilities from all points in the network is evaluated here. For a real assessment of access to facilities, it seems more interesting to perform an examination of the full admission of patients from emergency call to arrival in an CSC facility.

### Potential accessibility according to the type of patient intake procedure: complete approach from emergency call to the nearest CSC

Analysing accessibility at all points in the network of each stroke patient admission facility is an approach that has already been studied [16, 18, 19, 24], but with no allowance for different traffic scenarios. The analysis described here is innovative as—for the first time—it covers overall admission time starting with the emergency call and depending on the type of transport and traffic conditions. A great majority of stroke patients are transported by the fire brigade [4]. There are more fire stations than SMUR emergency units in the territory concerned. From the purely theoretical point of view and assuming that all fire stations and SMUR units are available, accessibility has been compared according to the mode of transport.

It is reminded that fire brigade access capability is greater than that of the SMUR medical teams. This greater density explains why potential access to patients by firemen and the admission of the formers to care facilities is greater than that of the SAMU, whatever the scenario. Thus the median access time by fire brigades is the best, as it is the average time. When speed limits are respected, median time for the firemen is 40.9 min and average time is 45.7 min from access to admission to the closest CSC facility, in comparison with a median of 45.9 min and an average of 55.9 min for the SAMU.

**Fig. 11:**
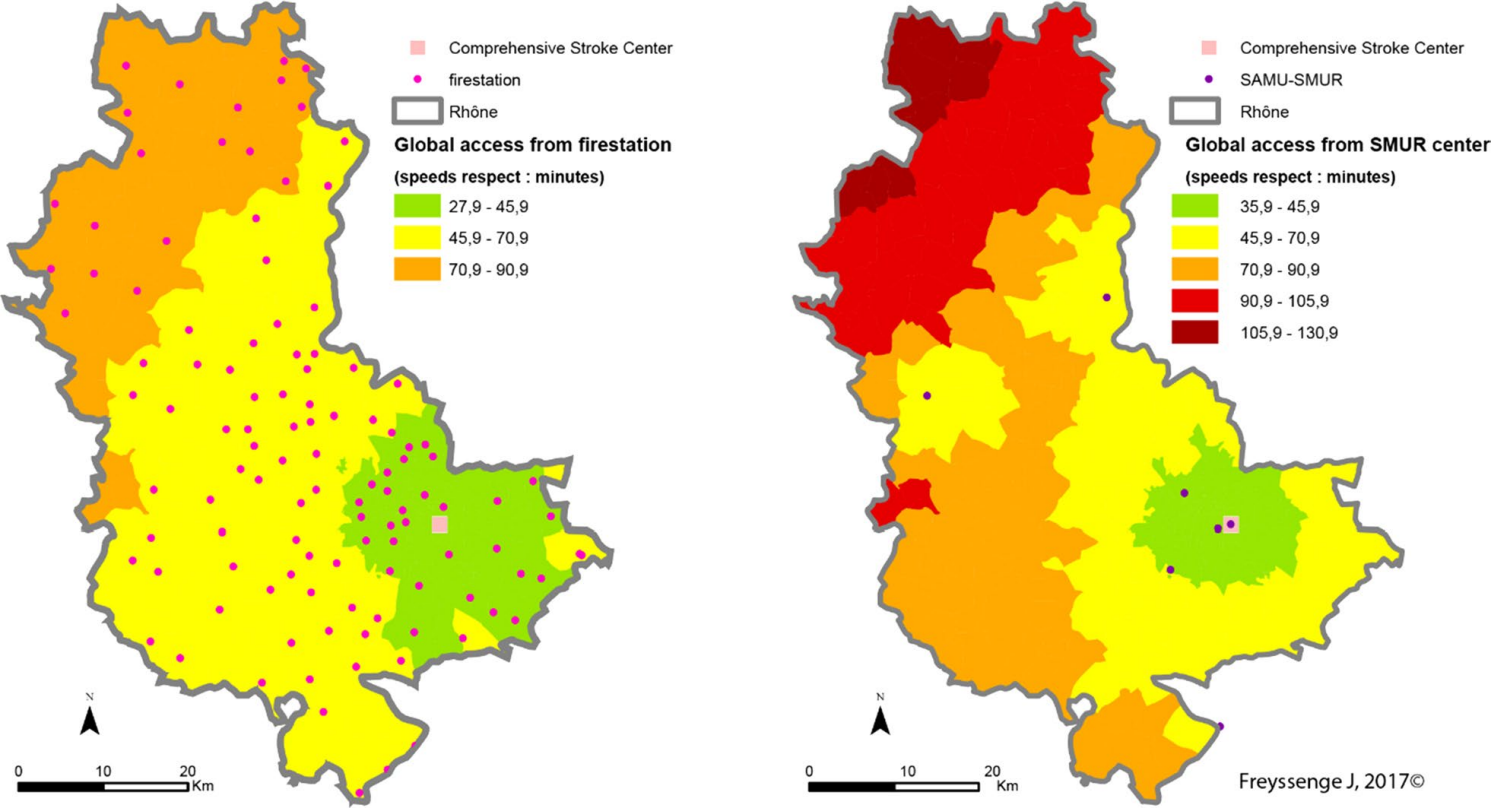
CSC admission times from a 15 emergency call (Scenario 1: speed limits respected) depending on the type of transport (on the left: fire brigade; on the right: SMUR)

As regards the territory, the towns at the northern extremity of the county display the most marked deficit in case of transport by the SMUR rather than the fire brigade, should every fire station be available. Thus stroke victims in the town of Monsols are between 1 h 45 min and 2 h 10 min from the CSC when transported by the SAMU and between 1 h 10 min and 1 h 30 min with the fire brigade—a gain of between 15 min and 1 h when speed limits are respected (Fig. 11). Admission times to CSC care are greatest in these areas that are the farthest from SMUR centres.

**Fig. 12:**
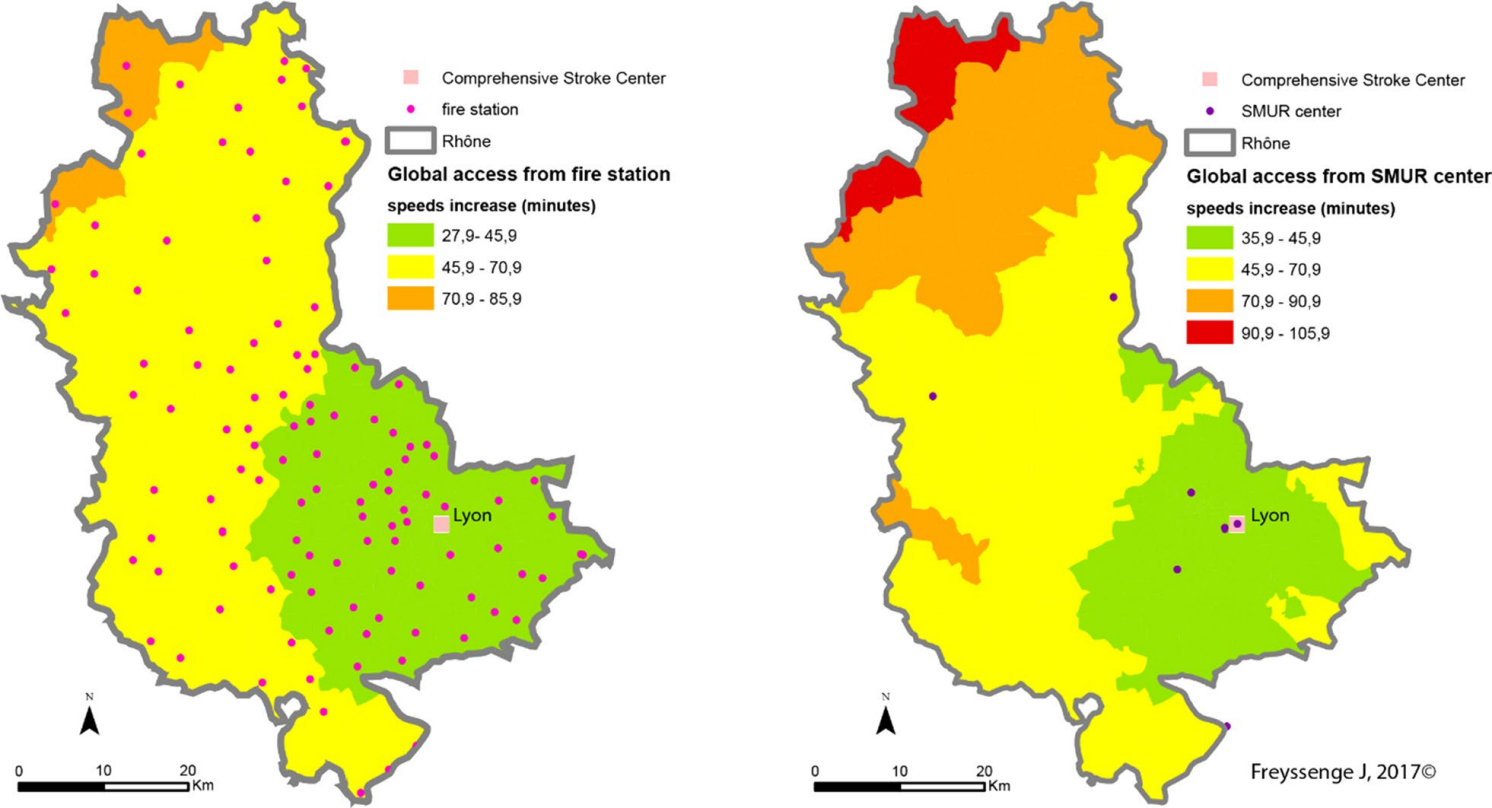
CSC admission periods after a 15 emergency call (Scenario 2—exceeding speed limits) depending on the transport mode (left: fire brigade; right: SMUR emergency service)

**Fig. 13:**
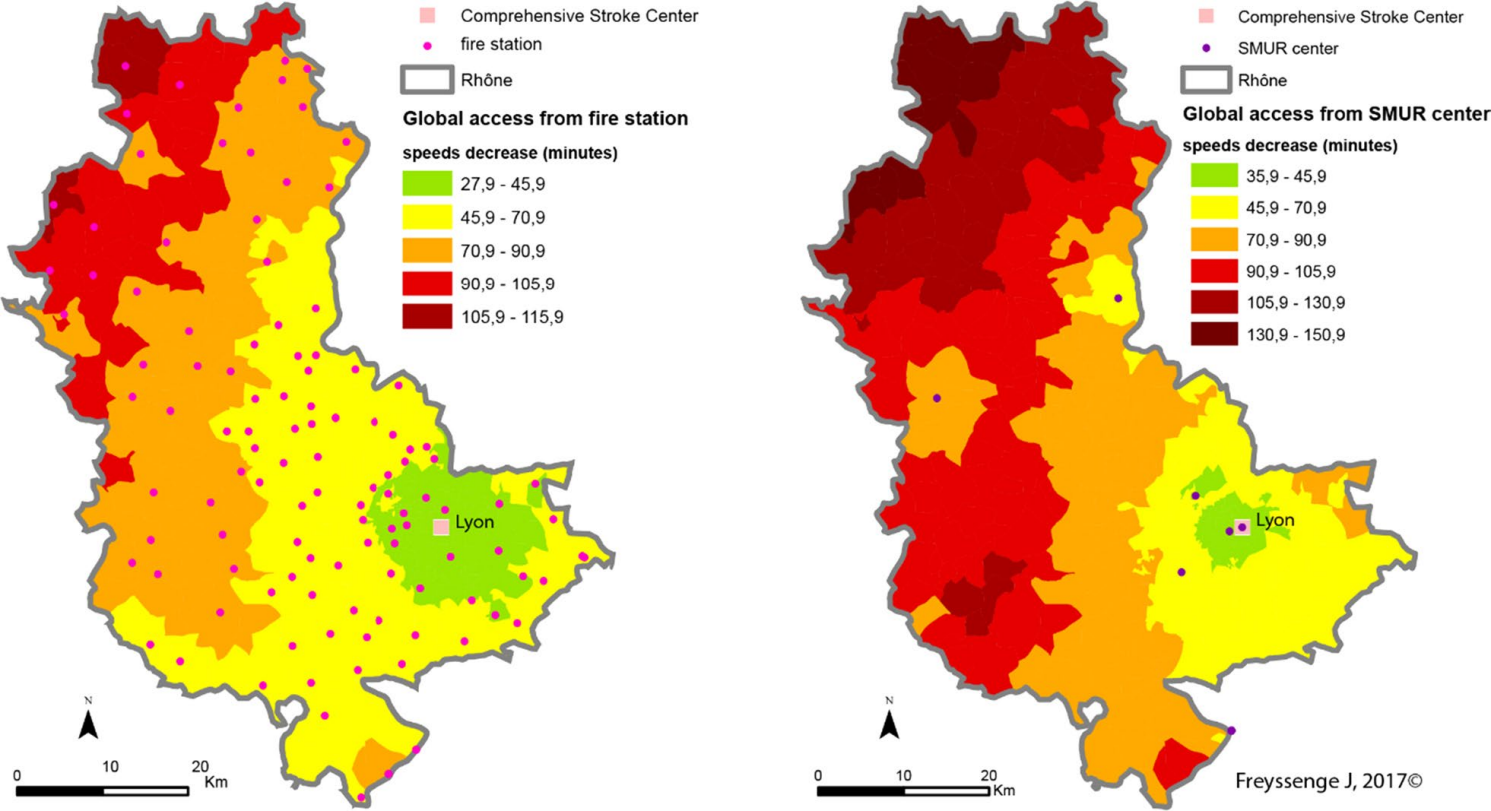
CSC admission periods are a 15 emergency call (Scenario 3—reducing speed limits) depending on the transport mode (left: fire brigade; right: SMUR emergency service)

The pattern is the same when speed limits are raised (Fig. 12) or lowered (Fig. 13). However, several features of individual scenarios should be noted.

Increasing the limits by 20 kph (12 mph) highlights the influence of the Lyons metropolis on the territory. The density of facilities—fire stations, SMUR units and CSC—here accounts for the marked influence of the Lyons urban area. Here, whatever the type of transport, patients are less than 45 min from admission to an CSC facility. In addition, in most of the territory increasing the speed limits by 20 kph can result in the patient gaining 15 min in CSC admission.

Finally, the main observation about reducing speed limits by 20 kph concerns the question of the eligibility of the patient for treatment. Travelling time in the northern most part of the Rhône county is 2 h with the firemen and possibly more with the SMUR. Without allowing for inter-hospital time but only times between the onset of symptoms and the 15 (emergency) call at between 15 min and 5 h [43], most of the patients in these areas are no longer eligible for thrombectomy.

## Discussion

The prime interest of the study is to model various scenarios based on different transport speeds. These multiple speeds are based on a review of the literature and on SAMU interventions.

This study has some practical implications. The different kind of transport are described and modelled. This modelling relies on transport times based on measured times that allows the comparison, for equivalent care conditions, between three kinds of transportation: private car and ambulances (firemen ambulance or SAMU ambulance) triggered by SAMU.

Classically, the accessibility is measured from patient’s care place to the nearest facility. This problematic has been analysed in this study, but the model has been more developed. Like it is observed in real case of care, the patient cannot go to the facility with his own mean. In a real context, the patient depends on modal transport and the access times linked to his care are from the trigger of transport care to his admission in a facility. The model presented gives a response to these care conditions.

Another practical implication is the emergence of territorial disparities. The measurement of access times to stroke patients allows to bring to light areas where we observe an extension of access delays to treatment.

As the method has been set out clearly, the finesses of modelling should now be improved. For example, ‘travel impactors’ [7] were not taken into account. These are in particular traffic lights and signs that lower the average speed of the vehicles carrying patients. The question of taking these impactors into account is raised especially for the modelling of patients travelling in their own vehicles.

Another limit but that is also a strong point of the study is the method used to know the maximum permitted speed in each section. *Route500*, the *Institut Géographique National* database commonly used in France for the various studies of this kind makes possible to use the Odomatrix program [44] to calculate travel time. Route 500 contains only 500,000 kilometres of the French road network. Furthermore, the maximum authorised speeds in each section are obtained for the category of each section of road (motorway, main road, regional link, local service) and the geographic environment traversed (urban, rural), which might not be truly representative. Indeed, numerous local features can be related to the setting of a speed limit. It was therefore chosen here to use the *BDCarto* database for its considerable exhaustiveness as it covers more than 1 million kilometres of road network. Although the use of this database is essential for the best possible analysis of the territory and to be as close to reality as possible, no speed limits are attributed to the sections. It was therefore necessary to use a participative facility—*Open Street Map* in this case—to model access times. This type of information can be one of the limits of the study. It is therefore planned to acquire a professional database such as Garmin^®^ or TomTom^®^ to gain accuracy in modelling.

The various modelling takes into account the accessibility of the territory at all points in the road network. The improvement of modelling would require integration of the time taken by emergency teams from the road network to the patient’s door. The question of accessibility is raised in particular for large housing block areas and residential zones where access to the patient’s door from the road network may require time in the light of the complex configuration of buildings and in particular blocks with no lifts.

Another note is about the required average time at the time regulation for decision-making and intervention average time. In the study, we use the set times by Adeoye et al. [19], from the US Emergency Medical Service (EMS) register. From one country to another, and from one region to another, emergency medical services organization varies greatly [45]. It’s therefore essential to estimate this times thanks to the SAMU registry. Thus, the models realized will be as close as possible to real life.

The modes of transport, firemen vs EMS, were compared is this study. This comparison was done to validate the method. In fact, in most cases, stroke patients are transported to hospital by firemen because they don’t need medical transport. Given the proportion of interventions, this comparison doesn’t have a high interest. Furthermore, the fire stations representation, which reflects the faster response of firemen, must be put into perspective. In fact, the future models should highlight the fire stations capacity to intervene. Apart from the highly-urbanized areas like Lyon and his urban agglomeration, firemen in rural fire stations are mostly volunteers. For this reason, if the nearest fire station doesn’t have the sufficient number of firemen, they can’t intervene and manage stroke patient which would extend access delays. The modelling of fire station ability to intervene will be feasible by representing their capacity according to the moment of the day, day/night in particular.

## Conclusions

This study is innovative and allows a characterization of the territory in term of potential accessibility by network and localization of every mode of transport, in term of patient location. In this case, the comparison between EMS (SMUR) and firemen transport will be improved, yet the study shows the capacities of GIS. Only access delays for a patient to healthcare structure are evaluated, the medical aspect linked to the patient psychological state at the moment of his management is not studied.

These different models are a method of decision making for healthcare organization. The use of our method and models as a complementary tool for regulation could be the subject of future studies.

Furthermore, this study is also innovative because the transport care is estimated as a whole, from 15 call to admission in CSC. With this kind of model, it’s easier to estimate the patient eligibility to thrombolysis and thrombectomy, with assumption of quickly recognizing of signs by the patient or people in his environment.

## Abbreviations

EMS: Emergency medical service
SAMU: Service d’Aide Médicale Urgente
SDIS: Service Départemental d’Incendie et de Secours
GDP: Gross domestic product
GIS: Geographic information systems
IGN: Institut National de l’information Géographique et forestière
Insee: Institut National de la Statistique et des Etudes Economiques
IRIS: Ilots Regroupés pour l’Information Statistique
SMUR: Structure Mobile d’Urgence et de Réanimation
PSC: Primary Stroke Center
CSC: Comprehensive Stroke Center
UMH: Unité Mobile Hospitalière
WHO: World Health Organization
